# Influence of drugs on blood potassium levels in older, multi-medicated patients – results of two cohort studies focusing on adverse drug reactions

**DOI:** 10.1186/s12877-026-07899-5

**Published:** 2026-06-27

**Authors:** Thea Laurentius, Esmail Al-Musawer, Katrin Schmitz, Cornelius Bollheimer, Justyna Wozniak, Verena Graeff, Andrea Kriegisch-Stumpf, Anja Knüppel-Ruppert, Harald Dormann, Julia C. Stingl, Katja S. Just

**Affiliations:** 1 Department of Geriatric Medicine, University Hospital RWTH Aachen, Aachen, Germany; 2 Institute of Clinical Pharmacology, University Hospital RWTH Aachen, Wendlingweg 2, Aachen, D-52074 Germany; 3 Central Emergency Department, University Hospital Augsburg, Augsburg, Germany; 4 Central Emergency Department, Hospital Ingolstadt, Ingolstadt, Germany; 5 Central Emergency Department, Hospital Fürth, Fürth, Germany

**Keywords:** Polypharmacy, Potassium, Drug-drug interaction, Renal function, Older adults, Geriatric patients, Quantile regression

## Abstract

**Background:**

Many drugs influence blood potassium levels and are often prescribed to older adults. Aim of this study was to estimate the extend of potassium level changes of drugs in the case of older, multi-medicated patients respecting renal function.

**Methods:**

Data of patients from an outpatient clinic (polypharmacy consultations hours) at the University Hospital RWTH Aachen and from a prospective, multicenter study on adverse drug reactions in emergency departments (ADRED) was used. Data of patients aged ≥ 70 years taking ≥ 3 drugs with available blood potassium levels were included. Quantile regression models were used to analyze drug effects in the context of multi-medication.

**Results:**

In total, *N* = 1097 patient cases were included with hypokalemia in 28.4% (*n* = 311) and hyperkalemia in 20.3% (*n* = 223). Male sex, use of low-ceiling diuretics, loop diuretics, mineralocorticoid receptor antagonists (MRAs), angiotensin-converting-enzyme inhibitors/ angiotensin II receptor blockers, number of other drugs, and CKD stage were associated with serum potassium levels and included in quantile regression models. In CKD stages 1 and 2, the use of MRAs (+ 0.49, *p* = 0.002), together with low-ceiling diuretics (-0.54, *p* < 0.001) had highest impact on serum potassium levels. In CKD stage 3, use of low-ceiling diuretics (-0.52, *p* < 0.001) and in CKD stages 4 and 5, the use of MRAs (+ 0.86, *p* < 0.001) had highest impact on serum potassium levels.

**Conclusions:**

Hypo- as well as hyperkalemia occur frequently in geriatric, multi-medicated patients. While renal function is an important predictor of blood potassium levels, drug effects can differ per drug class in the context of multi-medication.

**Trial registration:**

The ADRED-study is registered at the German Clinical Trials Register under: DRKS00008979. The cohort study of the polypharmacy consultation hours at the University Hospital RWTH Aachen is registered at the ClinicalTrials.gov under: NCT05247814.

**Supplementary Information:**

The online version contains supplementary material available at 10.1186/s12877-026-07899-5.

## Background

Many drugs influence blood potassium levels such as diuretics or antihypertensives and can, depending on the drug, cause both hypo- and hyperkalemia [1]. Those drugs influencing potassium levels are often prescribed to older adults as the prevalence of diseases such as heart failure and hypertension rises with age [2, 3]. Next to that, manifold factors and diseases, such as diabetes, cardiovascular diseases perspiration, diarrhea, vomiting, acidosis, or severe trauma can be associated with hypo- and hyperkalemia [4]. In addition, in old age a treatment with drug combinations is commonly needed either to control sufficiently the underlining disease or due to a combination of underlining diseases that necessitate a drug treatment with a combination of drugs [3]. Hence drug-drug interactions (DDI) are expected to occur often in older, multi-medicated adults [5]. DDIs can be harmful and lead to adverse drug reactions (ADRs) [6]. In the case of potassium levels, additive effects of drugs may cause hypo- or hyperkalemia, that both can lead to arrhythmias and even get life-threatening [7, 8]. This risk for arrhythmias is expected to increase in older, multi-medicated adults due to DDIs like for example in the case of digoxin given patients with hypo- or hyperkalemia [9].

DDIs are mostly analyzed pairwise in in vitro and in vivo studies, however in clinical settings several drug combinations can occur [10]. Analyzing DDIs is challenging as drug combinations occur relatively rarely and different mechanisms such as pharmacokinetics and –dynamics need to be respected. Therefore, the use of informatics was proposed years ago and is increasingly being implemented in methods for DDI analysis [11, 12]. Next to this, software solutions integrated in the electronic health record system or as standalone have existed for years, that can generate warnings on DDIs. However, DDI software typically analyzes only drug pairs and often generates excessive alerts by issue warnings that do not consider the patient’s overall condition [13]. As an example, an older adult with heart failure may be treated with a mineralocorticoid receptor antagonist (MRA) in combination with an angiotensin-converting-enzyme (ACE) inhibitor following treatment guidelines [14]. This commonly leads to warnings on an increased risk for hyperkalemia as both drug groups are known to increase potassium levels. This even happens if the same patient receives a loop diuretic, which is highly likely in the case of heart failure and is known for causing decreasing potassium levels [14]. However, it is not clear to what extend drugs, that increase or decrease potassium levels, interact in a specific patient.

This is increasingly important in older adults, as those patients get commonly treated with many drugs known to decrease or increase potassium levels. The prevalence of cardiovascular diseases is high among older adults, which increases the risk of sudden cardiac death in the cases of abnormal potassium levels [9]. In addition, kidney function is important for potassium homeostasis and may decrease in function during aging [15, 16].

Aim of this study was to estimate the extend of potassium level changes of drugs in the case of older, multi-medicated patients respecting renal function.

## Methods

### Study population and study design

A harmonized dataset of two cohorts of multi-medicated, older adults was analyzed, namely of the ADRED-study and a dataset of polypharmacy consultation hours at the University Hospital RWTH Aachen.

The ADRED-study was a prospective, multi-center observational study that collected patient cases between 2015 and 2021, that presented to six hospital emergency departments with symptoms rated as ADRs after standardized causality assessment [17, 18]. From previous analyses it is known that the ADRED-study covers a cohort of mostly older patients with high drug intake [18, 19].

The polypharmacy consultation hours at the University Hospital RWTH Aachen are offered to in and out-patients aged 70 years and older, that take a minimum of three drugs. Only first visits to consultation hours between May 2022 and September 2023 were respected for analysis (baseline visits). Comparably, cases of the ADRED-study, that were at least 70 years old and took at least three drugs were included in the analysis. All patients agreed in participation in the study and provided written informed consent. We combined the two datasets aiming to have less extreme potassium levels than expected when only analyzing cases of ADRs and increasing numbers of cases with normokalemia.

The ADRED-study was approved by the responsible ethical committee of the University of Bonn (202/15, trial registration: DRKS00008979). Collection of data from the polypharmacy consultation hours was approved by the ethical committee of RWTH Aachen University (393/21, trial registration: NCT05247814).

### Preparation of data

**Fig. 1:**
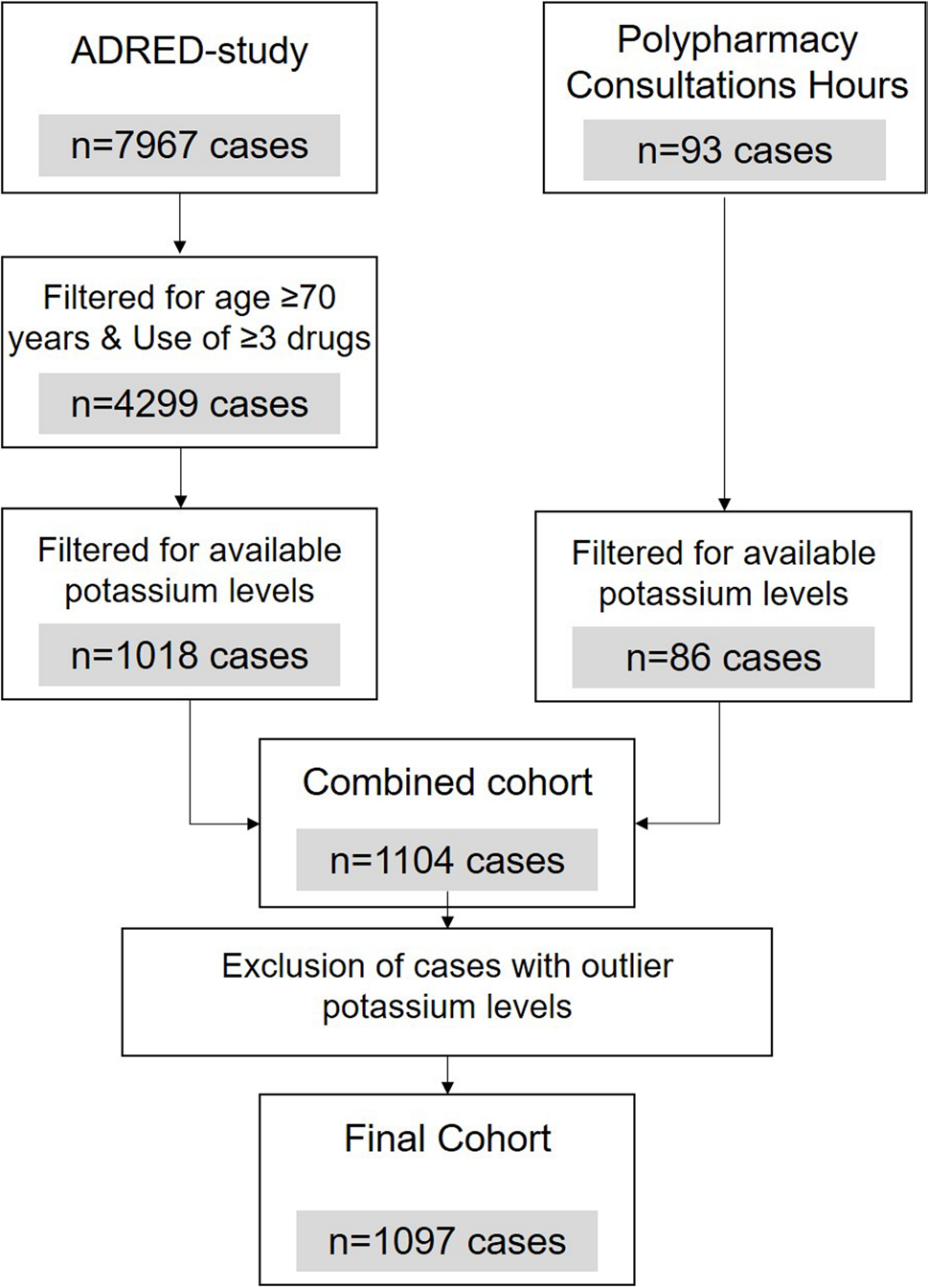
Flow chart presenting data harmonization of two cohorts analyzed. The ADRED-study was an observational study enrolling cases of adverse drug reactions in six emergency departments in Germany between 2015 and 2021. Patients presenting to the polypharmacy consultation hours at the University Hospital RWTH Aachen between May 2022 and June 2023 were included in data analysis

Both datasets were filtered for patients with documented potassium levels (Fig. 1). Demographic data (age and sex), status of treatment (out- or inpatient), and, where available, glomerular filtration rates (GFR) were calculated using the CKD-EPI formula based on serum creatinine levels [20]. Basing on estimated GFRs, stages of chronic kidney disease (CKD) were defined (stage 1 ≥ 90, stage 2 60–89, stage 3 30–59, stage 4 15–29, stage 5 < 15 mL/min/1.73m^2^). The number of documented diseases that were coded with ICD-10 codes in past medical history, was counted [21]. We grouped patients according to diagnoses of interest [4]: acute renal failure (N17) as the reason for hospitalization, a history of CKD (N18), history of dependence on renal dialysis or care involving dialysis (Z99.2, Z49), an acquired absence of kidney (Z90.5), heart failure (I50), chronic ischemic heart disease (CHD) (I25), essential hypertension (I10), atrial fibrillation or flutter (A-fib) (I48), and type 2 diabetes mellitus (T2DM) (E11). In addition, we analyzed patient reported symptoms on presentation basing on preferred terms following the Medical Dictionary for Regulatory Activities including those to be associated with hypo- or hyperkalemia and available in the dataset, namely: dizziness, palpitations, fatigue, diarrhea, obstipation, confusion, agitation, perspiration [22].

All drugs were documented including over the counter drugs and defined using the Anatomical Therapeutic Chemical (ATC) classification system [23]. The number of drug exposure was counted and documented per patient case. Drugs that are known to increase or decrease potassium levels were classified using ATC codes. A list of respective ATC codes and drug names that were documented in the two respective datasets can be found in the supplement (Supplement 1). Both datasets were merged into one dataset that was used for further analysis.

### Potassium levels

Serum potassium level distribution in the dataset was analyzed. Potassium levels ranged from 1.50 to 118.00 mmol/L. As tipping errors or collection errors when taking blood samples were suspected, outliers were removed from the analysis. Therefore, clinical not plausible values > 8.00 mmol/L were excluded. In a next step the dataset was checked for remaining outliers using the median absolute deviation method. Modified z-scores were calculated, and the dataset checked for values with an absolute score > 3.50 presenting potential remaining outliers. Thus, seven extreme cases with hyperkalemia were excluded from the analysis. Groups of patients with hypo-, normo- and hyperkalemia were formed. Because we aimed to analyze a geriatric cohort expected with high prevalence of hypertension and cardiac diseases [9], hypokalemia was defined up to a serum potassium level of 3.49 mmol/L, normokalemia as 3.50 mmol/L to 5.00 mmol/L, and hyperkalemia starting at 5.01 mmol/L.

### Statistical analysis

Descriptive analysis of the study cohort was conducted comparing patient cases with hypo-, normo-, and hyperkalemia. For continuous parameters median and interquartile ranges (IQR), and for categorical parameters absolute numbers and percentages were calculated. For univariate analysis, continuous parameters were compared between groups using Kruskal Wallis test, and categorical using Mantel Haenszel testing for a linear trend.

Quantile regression was performed estimating serum potassium 50% (median, Q50), and 25% (Q25), and 75% (Q75, hence IQR) in a stepwise approach including drug groups associated with a *p* ≤ 0.1 in univariate analysis, in addition to age, sex, and number of documented drugs. Patients under dialysis were excluded from this analysis (*n* = 21). The final model was adjusted for sex (male/ female), use of low-ceiling diuretics (thiazides or other low-ceiling diuretics), loop diuretics, MRAs, ACE inhibitors/ angiotensin II receptor blockers (ARBs) (all yes/ no), study cohort, and the number of drugs minus the drugs already included in the model. Specific effects were plotted with prediction lines. Analyses were repeated with subgroups including only patients with a GFR ≥ 60 mL/min/1.73m^2^ (CKD stage 1 and 2), GFR 30–59 mL/min/1.73m^2^ (CKD stage 3), and a GFR ≤ 29 mL/min/1.73m^2^ (CKD stage 4 and 5). We grouped CKD stages for sufficient sample sizes. In addition, we repeated all analyses in single CKD stages 1–5. Differences (delta, ∆) in predictions of potassium levels were calculated for Q50, Q25, and Q75 (e.g. predicted potassium level with use of loop diuretics – predicted potassium level without use of loop diuretics). To produce plots of quantile regression coefficients, the quantreg package in R (R Foundation for Statistical Computing, Vienna, Austria) was used. Regression coefficients, standard errors and 95% confidence intervals were obtained using nonparametric bootstrap resampling with 500 bootstrap replications. Regression coefficients and their corresponding 95% confidence intervals were extracted for each quantile and visualized using the ggplot2 package in R. Statistical analyses were conducted using IBM^®^ SPSS^®^ Statistics (version 28).

## Results

In total, *N* = 1097 patient cases were included in the analysis. The median potassium level in the cohort was 4.2 (IQR 3.4; 4.9). Of those 28.4% (*n* = 311) had low (median 3.1 mmol/L (IQR 2.9; 3.3)), 51.3% (*n* = 563) normal (4.3 (3.9; 4.6)), and 20.3% (*n* = 223) high potassium levels (5.5 (5.2; 6.0)). Descriptive characteristics of the study population are shown in Table 1 and descriptive characteristics stratified to the two datasets are shown in supplement 2.

**Table 1:** Descriptive characteristics of the study population (*N* = 1,097) comparing patient cases with low, normal and high serum potassium levels

	Missing, *n* (%)	Potassium ≤ 3.49 mmol/L, *n* = 311	Potassium 3.50–5.00 mmol/L, *n* = 563	Potassium ≥ 5.01 mmol/L, *n* = 223	*p*-value
Age (years), median (IQR)	-	80 (76; 85)	80 (75; 85)	79 (75; 84)	0.279
70–74 yrs, *n* (%)		57 (18.3)	132 (23.4)	45 (20.2)	
75–79 yrs, *n* (%)		78 (25.1)	141 (25.0)	70 (31.4)	
80–84 yrs, *n* (%)		95 (30.5)	146 (25.9)	58 (26.0)	
85–89 yrs, *n* (%)		53 (17.0)	104 (18.5)	32 (14.3)	
≥ 90 yrs, *n* (%)		28 (9.0)	40 (7.1)	18 (8.1)	
Sex (male), *n* (%)	-	131 (42.1)	274 (48.7)	135 (60.5)	**< 0.001**
Number of drugs, median (IQR)	-	9 (6; 12)	9 (7; 12)	10 (8; 13)	**0.001**
Number of comorbidities, median (IQR)	12 (1.1)	10 (7; 15)	10 (6; 15)	12 (8; 16)	**< 0.001**
Heart failure, *n* (%)		61 (19.9)	109 (19.6)	62 (27.8)	**0.043**
CHD, *n* (%)		67 (21.8)	162 (29.2)	102 (45.7)	**< 0.001**
Hypertension, *n* (%)		200 (65.1)	377 (67.9)	156 (70.0)	0.234
A-fib, *n* (%)		122 (39.7)	216 (38.9)	109 (48.9)	0.055
T2DM, *n* (%)		60 (19.5)	157 (28.3)	69 (30.9)	**0.002**
Absence of kidney, *n* (%)		5 (1.6)	3 (0.5)	4 (1.8)	0.976
Dialysis, *n* (%)		1 (0.3)	7 (1.3)	13 (5.8)	**< 0.001**
Acute renal failure, *n* (%)		18 (5.8)	34 (6.0)	44 (19.7)	**< 0.001**
CKD, *n* (%)		82 (26.7)	148 (26.7)	106 (47.5)	**< 0.001**
GFR (mL/min/1.73m^2^), median (IQR)	76 (6.9)	50.1 (33.6; 66.6)	47.7 (32.8; 65.2)	26.6 (15.9; 38.0)	**< 0.001**
Stages of chronic kidney disease based on GFR					
Stage 1, ≥ 90, *n* (%)		12 (4.2)	14 (2.6)	2 (1.0)	
Stage 2, 60–89, *n* (%)		81 (28.6)	163 (30.3)	9 (4.5)	
Stage 3, 30–59, *n* (%)		134 (47.3)	252 (46.8)	71 (35.5)	
Stage 4, 15–29, *n *(%)		41 (14.5)	82 (15.2)	73 (36.5)	
Stage 5, < 15, *n* (%)		15 (5.3)	27 (5.0)	45 (22.5)	
Patient reported symptoms	-				
Dizziness, *n* (%)		53 (17.0)	100 (17.8)	30 (13.5)	0.331
Palpitations, *n* (%)		9 (2.9)	21 (3.7)	4 (1.8)	0.568
Fatigue, *n* (%)		7 (2.3)	33 (5.9)	12 (5.4)	0.060
Diarrhea, *n* (%)		42 (13.5)	44 (7.8)	9 (4.0)	**< 0.001**
Obstipation, *n* (%)		18 (5.8)	24 (6.0)	9 (4.0)	0.435
Confusion, *n* (%)		17 (5.5)	12 (2.1)	4 (1.8)	**0.008**
Agitation, *n* (%)		13 (4.2)	23 (4.1)	4 (1.8)	0.179
Perspiration, *n* (%)		1 (0.3)	12 (2.1)	3 (1.3)	0.233

*IQR *interquartile ranges, *CHD *chronic heart disease, *A-fib *atrial fibrillation, *T2DM *type 2 diabetes mellitus, *CKD *chronic kidney disease, *GFR *glomerular filtration rate

Significant findings in bold text

Males were observed more often with high potassium levels. Patients with hyperkalemia had frequently a higher stage of CKD and more documented comorbidities and were known more often for a history of heart failure, CHD, A-fib, and T2DM. Patients with hyperkalemia presented more often with acute renal failure, had a history of CKD, and had a known dependence on dialysis. Patients that presented with hypokalemia complaint more often about diarrhea and were confused on presentation.

Several drugs expected to cause low potassium levels, such as low-ceiling diuretics including thiazides or other low-ceiling diuretics, were commonly found more often in patients with low potassium levels in univariate analysis. In contrast, loop diuretics were more frequently observed in patients with high compared to low potassium levels. This was likewise seen for systemic glucocorticoids. Distribution of drug exposure between potassium level groups is shown in Table 2 and stratified analysis in supplement 3. Insulins and osmotically acting laxatives seemed not associated with potassium levels in univariate analysis.

**Table 2:** Distribution of drug use in patients with low, normal and high potassium levels (*N* = 1,097)

Drugs	Potassium ≤ 3.49 mmol/l, *n* = 311	Potassium 3.50–5.00 mmol/l, *n* = 563	Potassium ≥ 5.01 mmol/l, *n* = 223	*p*-value
	Expected low potassium levels			
Low-ceiling diuretics, *n* (%)	94 (30.2)	77 (13.7)	26 (11.7)	**< 0.001**
Thiazides, *n* (%)	54 (17.4)	44 (7.8)	15 (10.3)	**< 0.001**
Other low-ceiling diuretics, *n* (%)	40 (12.9)	34 (6.0)	11 (4.9)	**< 0.001**
Loop diuretics, *n* (%)	173 (55.6)	294 (52.2)	144 (64.6)	0.079
Insulins, *n* (%)	36 (11.6)	86 (15.3)	36 (16.1)	0.116
Glucocorticoids, *n* (%)	38 (12.2)	53 (9.4)	39 (17.5)	0.124
Osmotically acting laxatives, *n* (%)	38 (12.2)	87 (15.5)	24 (10.8)	0.795
	*Expected high potassium levels*			
MRAs, *n* (%)	34 (10.9)	86 (15.3)	75 (33.6)	**< 0.001**
ACE inhibitors/ ARBs, *n* (%)	172 (55.3)	387 (68.7)	153 (68.6)	**< 0.001**
ACE inhibitors, *n* (%)	92 (29.6)	211 (37.5)	81 (36.3)	0.071
(ARBs), *n* (%)	81 (26.0)	178 (31.6)	74 (33.2)	0.062
Beta blocking agents, *n* (%)	195 (62.7)	382 (67.9)	166 (74.4)	**0.004**
NSAIDs, *n* (%)	22 (7.1)	48 (8.5)	20 (9.0)	0.408
Coxibs, *n* (%)	2 (0.6)	2 (0.4)	7 (3.1)	**0.010**
Potassium, *n* (%)	43 (13.8)	41 (7.3)	14 (6.3)	**0.001**

*ARBs *Angiotensin II receptor blockers, *MRAs *mineralocorticoid receptor antagonists, *NSAIDs *Non-steroidal anti-inflammatory drugs

Significant findings in bold text

Drugs expected to cause high potassium levels, such as MRAs, ACE inhibitors/ARBs, and beta-blockers, were observed more often in patients with high potassium levels. Non-steroidal anti-inflammatory drugs did not show a distribution to be more often in patients with high potassium levels, but coxibs did, however with low frequency (*n* = 11) and only in the ADRED-dataset. The use of potassium as a drug showed a reverse distribution with being more often in patients with low potassium.

**Fig. 2:**
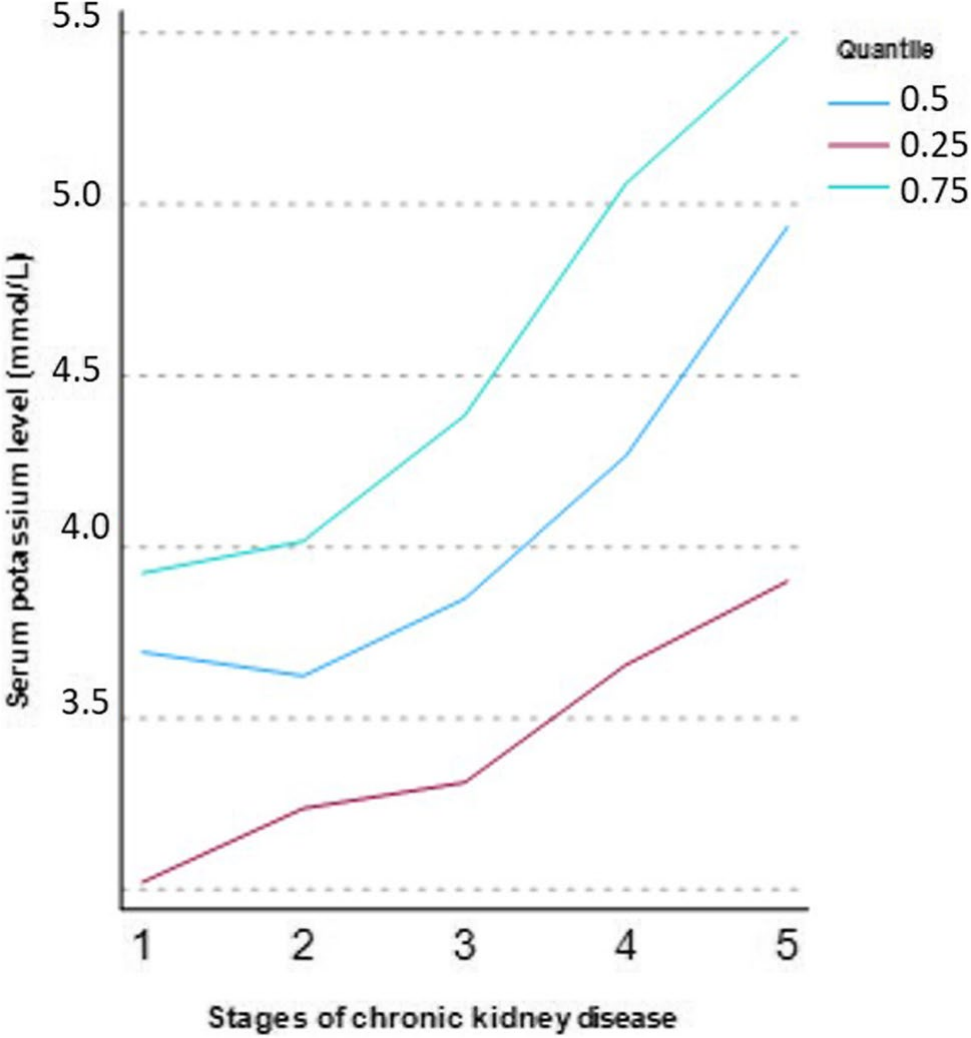
Prediction of serum potassium levels according to stages of chronic kidney disease defined by estimated glomerular filtration rates (GFR) using a quantile regression model. Model adjusted for sex, use low-ceiling diuretics, loop diuretics, MRAs, ACE inhibitors/ ARBs, and number of other drugs. ARBs: Angiotensin II receptor blockers; MRAs: mineralocorticoid receptor antagonists

Male sex, use of low-ceiling diuretics, loop diuretics, MRAs, ACE inhibitors/ARBs, number of other drugs, and CKD stage according to estimated GFR were significantly associated with serum potassium levels and hence included in quantile regression models. Beta blockers were not significantly associated with serum potassium levels and therefore not included in the final model. The CKD stage according to estimated GFR was found to be a strong predictor of serum potassium levels in the model (Fig. 2). Predictions of potassium levels ranged from stage 1 (GFR ≥ 90) 3.69 mmol/L (3.02; 3.92) to stage 5 (GFR < 15) 4.93 (3.90; 5.49). The use of loop diuretics, MRAs, and low-ceiling diuretics other than thiazides was more frequently observed with higher stages of CKD (Table 3). A similar trend could be observed for the use of ACE inhibitors/ARBs, while the use of thiazides became less common with higher CKD stages, although both not significant.

**Table 3:** Distribution of drug use stratified to stages of chronic kidney disease (CKD) based on glomerular filtration rates (GFR) (*N* = 1,001)

	CKD stage 1 & 2, GFR ≥ 60 ml/min/1.73m^2^, *n* = 281	CKD stage 3, GFR 30–59 ml/min/1.73m^2^, *n* = 456	CKD stage 4 and 5, GFR ≤ 29 ml/min/1.73m^2^, *n* = 264	*p*-value
Male sex, *n* (%)	133 (47.3)	228 (50.0)	129 (48.9)	0.711
Low-ceiling diuretics, *n* (%)	43 (15.3)	81 (17.8)	51 (19.3)	0.216
Thiazides, *n* (%)	30 (10.7)	44 (9.6)	21 (8.0)	0.280
Other low-ceiling diuretics, *n* (%)	13 (4.6)	37 (8.1)	30 (11.4)	**0.004**
Loop diuretics, *n* (%)	92 (32.7)	251 (55.0)	201 (76.1)	**< 0.001**
MRAs, *n* (%)	28 (10.0)	73 (16.0)	70 (26.5)	**< 0.001**
ACE inhibitors/ ARBs, *n* (%)	171 (60.9)	301 (66.0)	177 (67.0)	0.127
ACE inhibitors, *n* (%)	97 (34.5)	156 (34.2)	101 (38.3)	0.370
ARBs, *n* (%)	75 (26.7)	147 (32.2)	78 (29.5)	0.449

Patients with known dialysis were excluded from the analysis

*IQR *interquartile range, *ARBs *Angiotensin II receptor blockers, *MRAs *mineralocorticoid receptor antagonists

Significant findings in bold text

**Fig. 3:**
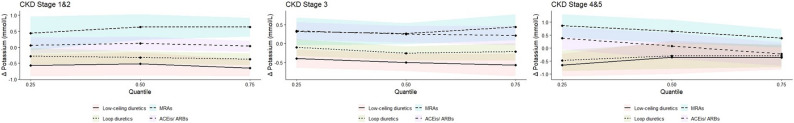
Estimation of drug effects on serum potassium levels (potassium ((mmol/L)) stratified in patients with chronic kidney disease (CKD) stage 1 and 2, CKD stage 3, and CKD stage 4 and 5. All models adjusted for sex, use of low-ceiling diuretics, loop diuretics, MRAs, ACE inhibitors/ ARBs, and number of other drugs. ARBs: Angiotensin II receptor blockers; MRAs: mineralocorticoid receptor antagonists

In patients with CKD stages 1 and 2, the use of MRAs, together with low-ceiling diuretics had highest impact on serum potassium levels, while the use of ACE inhibitors/ ARBs was not seen significantly associated (Table 4). In patients with CKD stage 3, use of low-ceiling diuretics had the highest impact on serum potassium levels. In patients with CKD stages 4 and 5, the use of MRAs had highest impact on serum potassium levels, while the use of the other drug groups did not consistently had a significant impact on serum potassium levels in all three quantiles in the model (Fig. 3). We observed comparable results, although with less significance due to sample sizes in single CKD stages groups (Supplement 4). The number of other drugs not included in the model, showed significant association with serum potassium levels in all CKD stages analyzed with more drugs indicating higher potassium levels.

**Table 4:** Predictions of changes of serum potassium levels (median Q50 (Q25; Q75)) in mmol/L stratified in stages of chronic kidney disease (CKD) according to estimated glomerular filtration rates (GFR) in multivariate quantile regression models

	CKD stage 1 & 2, GFR ≥ 60 ml/min/1.73m^2^, *n* = 281		CKD stage 3, GFR 30–59 ml/min/1.73m^2^, *n* = 456		CKD stage 4 and 5, GFR ≤ 29 ml/min/1.73m^2^, *n* = 264	
	∆Q50 (∆Q25; ∆Q75)	*p*-value per Q50 (Q25; Q75)	∆Q50 (∆Q25; ∆Q75)	*p*-value per Q50 (Q25; Q75)	∆Q50 (∆Q25; ∆Q75)	*p*-value per Q50 (Q25; Q75)
Male sex	0.35 (0.10; 0.31)	**< 0.001** (0.289; <**0.001**)	0.35 (0.37; 0.21)	**< 0.001 (< 0.001;** 0.056**)**	0.04 (0.22; 0.24)	0.279 (0.814; 0.151)
Low-ceiling diuretics	-0.54 (-0.55; -0.65)	**< 0.001 (< 0.001; <0.001)**	-0.52 (-0.35; -0.52)	**< 0.001 (0.007; <0.001)**	-0.45 (-0.63; -0.38)	0.078 (**0.003**; 0.069)
Loop diuretics	-0.30 (-0.26; -0.35)	**0.003 (0.013; <0.001)**	-0.26 (-0.10; -0.20)	**0.016** (0.335; 0.088)	-0.09 (-0.33; -0.28)	0.697 (0.090; 0.149)
MRAs	0.49 (0.43; 0.66)	**0.002 (0.007; <0.001)**	0.28 (0.34; 0.42)	0.050 (**0.013; 0.005**)	0.86 (1.01; 0.40)	**< 0.001 (< 0.001; 0.033)**
ACE inhibitors/ ARBs	0.13 (0.09; 0.07)	0.185 (0.367; 0.427)	0.23 (0.33; 0.25)	**0.035** (**0.001**; **0.034**)	0.36 (0.47; <0.01)	0.092 (**0.007**; >0.999)

Models adjusted for sex, use of low-ceiling diuretics, loop diuretics, MRAs, ACE inhibitors/ ARBs, study cohort, and number of other drugs

*ARBs *Angiotensin II receptor blockers, *MRAs *mineralocorticoid receptor antagonists

Significant findings in bold text

## Discussion

This study shows the differences in blood potassium levels between drug groups and renal function and allows, to the best of our knowledge for the first time, a ranking between drug effects in the case of multi-medication.

Concerning high potassium levels, MRAs as well as ACE inhibitors and ARBs are expected to cause hyperkalemia, but MRAs showed a significantly higher impact on potassium levels than ACE inhibitors and ARBs in our analysis. In the literature, MRAs are not expected to add a significant risk for hyperkalemia when ACE inhibitors or ARBs are added to the treatment [24]. Hyperkalemia is a well-known symptom in very old patients treated with MRAs [25]. While no distinct differences of influences on potassium levels could be observed between ACE inhibitors and ARBs, MRAs might increase the potassium level to a much higher extent. As ACE inhibitors and ARBs are a column of pharmacological heart failure therapy, in older, multi-medicated or even frail adults, MRAs might be used with more caution using a close monitoring [26].

On the other hand, low-ceiling diuretics showed higher association with low potassium levels in our analysis compared to high-ceiling diuretics, while the diuretic effect is expected to be the opposite. A combination of these drugs is rare but could be indicated to reduce the risk for death in patients with heart failure with reduced ejection fraction or with acute heart failure and volume overload [14, 27]. While low-ceiling diuretics show comparable effects in lowering blood potassium levels [28, 29], the potassium-lowering effects should be observed with caution, especially in the case of multi-medication.

The univariate analysis underlines the challenge to address drug effects adequately in multi-morbid and –medicated patients. As an example, loop diuretics are more frequently seen in patients with hyperkalemia in univariate analysis in our dataset, whereas multivariate analysis shows lower potassium levels. The higher frequency of loop diuretics might be connected to higher CKD stages which themselves are associated with higher potassium levels [30]. Notably, we calculated CKD stages based on GFR values at ED admission. These GFR values might not always present the baseline CKD stage, but are prone to urgent modifications due to the ADR leading to ED admission. In this line, the use of drugs such as MRAs or certain low-ceiling diuretics in CKD stages 4 and 5 need to be interpreted. We cannot distinguish whether medications were prescribed acutely or had already been given long-term before ED admission. However, it would be reasonable to assume that these drugs were ongoing medications while the renal function diminished. Our real-world data shows the importance and possible problems with drug use also in urgently occurring renal failure in the context of multi-medication. Notably, we focused our models on use of certain drug groups as the interplay with certain diseases is difficult to address. Hence, more potential confounding factors might be of relevance for such analyses. Our results underline the need for multivariate models established with pharmacological and clinical background to address potential confounders adequately.

The influence of drug groups on blood potassium levels has distinct differences, that need to be respected when treating older, multi-medicated patients. In older adults, there is a higher risk to present with dehydration, which can already make slight disturbances in renal function and potassium levels clinically relevant [31]. In addition, frailty might be associated with electrolyte disturbances [32]. Hence, focus should be laid on both hypo- and hyperkalemia as both might occur in older adults in the context of multi-medication.

Our study has some limitations. In addition to data of a single outpatient clinic, we used data of a prospective study on ADRs on emergency department admission, thereby potentially enrolling patients with high variability of potassium levels due to the acute presentation. We addressed this issue by excluding outliers from our analysis and using the dataset of the polypharmacy consultation hours. But still, there might be some cases of hyperkalemia resulting from difficulties in blood sampling as an example. Further, in particular in the ADRED-cohort several cases had no potassium levels documented. As documenting potassium levels was not obligatory in the ADRED-study, this is reasonable. While median and IQR values show a wide range of potassium levels in the normal range, a selection bias cannot fully be ruled out. Hence, the distinct changes in potassium levels resulting from quantile regression need to be generalized with caution. We tested in multivariate regression models the use of multiple drug groups, even though in some cases not all drug groups might be used at the same time and we cannot address uncertainty of compliance. In addition, we used a statistical selection approach for inclusion of certain drug groups (*p* > 0.1 in univariate analysis), thus potentially producing less generalizability. Yet, age, sex, and number of used drugs were used a priori as variables as confounding was likely. However, further confounding was not respected in the analysis due to the sample size and availability of data. Hence, remaining residual confounding is likely. Confidence intervals of analysis are not very large indicating an appropriate sample size. We included only some drug groups known to cause high or low potassium levels and by using this selective approach might have missed other drugs being likewise important. To address this, we included the number of other drugs used in the regression models, which was not significant. In addition, the drug groups we included are often found in older adults (e.g. in the context of heart failure and/or hypertension) and therefore clinically relevant.

Strength of our study is the focus on the older, multi-medicated patient who is often underrepresented in studies [33]. Additionally, DDIs are often addressed only in a bi-directional manner, and interactions involving multiple medications, including more than two drugs or drug groups are frequently not addressed appropriately [10].

In conclusion, hypo- as well as hyperkalemia might occur easily in geriatric, multi-medicated patients. MRAs and low-ceiling diuretics should be used with caution in these patients. While renal function is certainly an important predictor of blood potassium levels, drug effects might differ per drug class in the context of multi-medication. More studies analyzing DDIs in the context of multi-medication are needed.

## Supplementary Information

Supplementary Material 1.

Supplementary Material 2.

Supplementary Material 3.

Supplementary Material 4.

## Data Availability

The data that support the findings of this study are available from the corresponding author upon reasonable request.

## Abbreviations

ACE: Angiotensin-converting-enzyme
ADR: Adverse Drug Reaction
A-fib: Atrial Fibrillation or Flutter
ARB: Angiotensin II Receptor Blocker
ATC: Anatomical Therapeutic Chemical
CHD: Chronic Ischemic Heart Disease
CKD: Chronic Kidney Disease
DDI: Drug-drug Interactions
GFR: Glomerular Filtration Rates
IQR: Interquartile Ranges
MRA: Mineralocorticoid Receptor Antagonist
T2DM: Type 2 Diabetes Mellitus
