# Supplementary material for: Influence of drugs on blood potassium levels in older, multi-medicated patients – results of two cohort studies focusing on adverse drug reactions
Source: BMC Geriatr. 2026 Jun 27;26:881. doi: 10.1186/s12877-026-07899-5 (PMC13317038; doi:10.1186/s12877-026-07899-5)
Supplement: Supplementary file 1 — Supplementary Material 1. [file 12877_2026_7899_MOESM1_ESM.docx]

**Supplement 1**: List of drugs influencing potassium levels included in the study

| **Drug(group)** | **Subgroup** | **ATC-code** |
| --- | --- | --- |
| *Decreasing potassium level* | | |
| Thiazides |  | C03A |
|  | Thiazides in combination with potassium-sparing agents | C03EA |
|  | ACE inhibitors and diuretics* | C09BA |
|  | ARBs and diuretics* | C09DA |
|  | Beta blocking agents and thiazides | C07B |
| Other low-ceiling diuretics | Other low-ceiling diuretics, plain | C03BA |
|  | Beta blocking agents and other diuretics** | C07C |
| Loop diuretics | Loop diuretics, plain | C03C |
|  | Loop diuretics and aldosterone antagonists | C03ED |
| Insulins |  | A10A |
| Glucocorticoids | Only systemic use | H02 |
| Osmotically acting laxatives |  | A06AD |
| *Increasing potassium level* | | |
| Aldosterone antagonists and potassium-sparing agents |  | C03D |
|  | Loop diuretics and aldosterone antagonists | C03ED |
| ACE inhibitors | ACE inhibitors, plain | C09AA |
|  | ACE inhibitors and diuretics* | C09BA |
|  | ACE inhibitors and calcium channel blockers | C09BB |
|  | ACE inhibitors, other combinations | C09BX |
| Angiotensin II receptor blockers (ARBs) | ARBs, plain | C09C |
|  | ARBs and diuretics* | C09DA |
|  | ARBs and calcium channel blockers | C09DB |
|  | ARBs, other combinations | C09DX |
| Beta blocking agents | Beta blocking agents, plain | C07A |
|  | Beta blocking agents and thiazides | C07B |
|  | Beta blocking agents and other diuretics** | C07C |
| Non-steroidal anti-inflammatory drugs (NSAIDs) |  | M01AB  M01AC  M01AE |
|  | Coxibs | M01AH |
| Potassium |  | A12BA |

* in the groups of ACE inhibitors/ARBs and diuretics only thiazides are combined

** in the group of beta blocking agents and other diuretics only drugs including indapamide were listed in the dataset
