# Supplementary material for: Influence of drugs on blood potassium levels in older, multi-medicated patients – results of two cohort studies focusing on adverse drug reactions
Source: BMC Geriatr. 2026 Jun 27;26:881. doi: 10.1186/s12877-026-07899-5 (PMC13317038; doi:10.1186/s12877-026-07899-5)
Supplement: Supplementary file 2 — Supplementary Material 2. [file 12877_2026_7899_MOESM2_ESM.docx]

**Supplement 2.** Descriptive characteristics of the study population (N=1,097) comparing patient cases with low, normal and high serum potassium levels stratified to datasets.

|  | **ADRED-study, n =1011** | | | | | **Polypharmacy consultation hours, n=86** | | | | |
| --- | --- | --- | --- | --- | --- | --- | --- | --- | --- | --- |
|  | **Missing, n (%)** | **Potassium ≤ 3.49 mmol/L, n=306** | **Potassium 3.50 – 5.00 mmol/L, n=490** | **Potassium ≥5.01 mmol/L, n=215** | **p-value** | **Missing, n (%)** | **Potassium ≤ 3.49 mmol/L, n=5** | **Potassium 3.50 – 5.00 mmol/L, n=73** | **Potassium ≥5.01 mmol/L, n=8** | **p-value** |
| Age (years), median (IQR) | - | 80 (76; 85) | 79 (74; 84) | 79 (75; 84) | 0.080 | - | 77 (76; 87) | 83 (79; 87) | 82 (75; 85) | 0.560 |
| 70 – 74 yrs, n (%) |  | 57 (18.6) | 123 (25.1) | 43 (20.0) |  |  | 0 (0.0) | 9 (12.3) | 2 (25.0) |  |
| 75 – 79 yrs, n (%) |  | 75 (24.5) | 128 (26.1) | 70 (32.6) |  |  | 3 (60.0) | 13 (17.8) | 0 (0.0) |  |
| 80 – 84 yrs, n (%) |  | 94 (30.7) | 124 (25.3) | 54 (25.1) |  |  | 1 (20.0) | 22 (30.1) | 4 (50.0) |  |
| 85 – 89 yrs, n (%) |  | 53 (17.3) | 83 (16.9) | 30 (14.0) |  |  | 0 (0.0) | 21 (28.8) | 2 (25.0) |  |
| ≥ 90 yrs, n (%) |  | 27 (8.8) | 32 (6.5) | 18 (8.4) |  |  | 1 (20.0) | 8 (11.0) | 0 (0.0) |  |
| Sex (male), n (%) | - | 131 (42.8) | 257 (52.4) | 132 (61.4) | **<0.001** | - | 5 (100) | 56 (76.7) | 5 (62.5) | 0.131 |
| Number of drugs, median (IQR) | - | 9 (6; 12) | 9 (6; 12) | 10 (8; 12) | **<0.001** | - | 16 (13; 20) | 14 (10; 18) | 16 (13; 19) | 0.395 |
| Number of comorbidities, median (IQR) | 12 (1.2) | 10 (7; 14) | 10 (6; 14) | 12 (8; 16) | **<0.001** | - | 16 (12; 19) | 13 (8; 20) | 11 (9; 14) | 0.460 |
| Heart failure, n (%) |  | 59 (19.5) | 88 (18.3) | 61 (28.4) | **0.028** |  | 2 (40.0) | 21 (28.8) | 1 (12.5) | 0.257 |
| CHD, n (%) |  | 66 (21.9) | 136 (28.2) | 99 (46.0) | **<0.001** |  | 1 (20.0) | 26 (35.6) | 3 (37.5) | 0.580 |
| Hypertension, n (%) |  | 197 (65.2) | 322 (66.8) | 148 (68.8) | 0.393 |  | 3 (60.0) | 55 (75.3) | 8 (100) | 0.077 |
| A-fib, n (%) |  | 119 (39.4) | 180 (37.3) | 104 (48.4) | 0.070 |  | 3 (60.0) | 36 (49.3) | 5 (62.5) | 0.797 |
| T2DM, n (%) |  | 57 (18.9) | 135 (28.0) | 63 (29.3) | **0.004** |  | 3 (60.0) | 22 (30.1) | 6 (75.0) | 0.269 |
| Absence of kidney, n (%) |  | 5 (1.7) | 3 (0.6) | 4 (1.9) | 0.985 |  | 0 (0.0) | 0 (0.0) | 0 (0.0) | - |
| Dialysis, n (%) |  | 1 (0.3) | 7 (1.5) | 13 (6.0) | **<0.001** |  | 0 (0.0) | 0 (0.0) | 0 (0.0) | - |
| Acute renal failure, n (%) |  | 18 (5.9) | 34 (6.9) | 44 (20.5) | **<0.001** |  | 0 (0.0) | 0 (0.0) | 0 (0.0) | - |
| CKD, n (%) |  | 80 (26.5) | 123 (25.5) | 102 (47.4) | **<0.001** |  | 2 (40.0) | 52 (34.2) | 4 (50.0) | 0.596 |
| GFR (mL/min/1.73m^2^), median (IQR) | 75 (7.4) | 50.3 (33.9; 66.7) | 46.5 (31.4; 64.8) | 26.0 (15.7; 36.7) | **<0.001** | 1 (1.2) | 41.0 (25.5; 63.0) | 58.0 (42.0; 77.8) | 47.5 (44.3; 57;3) | 0.233 |
| Stages of chronic kidney disease based on GFR | | | | | |  |  |  |  |  |
| Stage 1, ≥ 90, n (%) |  | 12 (4.3) | 12 (2.6) | 2 (1.0) |  |  | 0 (0.0) | 2 (2.8) | 0 (0.0) |  |
| Stage 2, 60 - 89, n (%) |  | 80 (28.8) | 131 (28.1) | 8 (4.2) |  |  | 1 (20.0) | 32 (44.4) | 1 (12.5) |  |
| Stage 3, 30 - 59, n (%) |  | 131 (47.1) | 220 (47.2) | 65 (33.9) |  |  | 3 (60.0) | 32 (44.4) | 6 (75.0) |  |
| Stage 4, 15 - 29, n (%) |  | 40 (14.4) | 76 (16.3) | 72 (37.5) |  |  | 1 (20.0) | 6 (8.3) | 1 (12.5) |  |
| Stage 5, < 15, n (%) |  | 15 (5.4) | 27 (5.8) | 45 (23.4) |  |  | 0 (0.0) | 0 (0.0) | 0 (0.0) |  |
| Patient reported symptoms | - |  |  |  |  | - |  |  |  |  |
| Dizziness, n (%) |  | 50 (16.3) | 74 (15.1) | 26 (12.1) | 0.192 |  | 3 (60.0) | 26 (35.6) | 4 (50.0) | 0.931 |
| Palpitations, n (%) |  | 7 (2.3) | 7 (1.4) | 2 (0.9) | 0.208 |  | 2 (40.0) | 14 (19.2) | 2 (25.0) | 0.669 |
| Fatigue, n (%) |  | 3 (1.0) | 7 (1.4) | 7 (3.3) | 0.058 |  | 4 (80.0) | 26 (35.6) | 5 (62.5) | 0.901 |
| Diarrhea, n (%) |  | 41 (13.4) | 35 (7.1) | 9 (4.2) | **<0.001** |  | 1 (20.0) | 9 (12.3) | 0 (0.0) | 0.244 |
| Obstipation, n (%) |  | 16 (5.2) | 13 (2.7) | 5 (2.3) | 0.052 |  | 2 (40.0) | 21 (28.8) | 4 (50.0) | 0.528 |
| Confusion, n (%) |  | 17 (5.6) | 6 (1.2) | 4 (1.9) | **0.004** |  | 0 (0.0) | 6 (8.2) | 0 (0.0) | 0.820 |
| Agitation, n (%) |  | 9 (2.9) | 4 (0.8) | 2 (0.9) | **0.039** |  | 4 (80.0) | 19 (26.0) | 2 (25.0) | 0.080 |
| Perspiration, n (%) |  | 1 (0.3) | 1 (0.2) | 2 (0.9) | 0.339 |  | 0 (0.0) | 11 (15.1) | 1 (12.5) | 0.642 |

IQR: interquartile ranges; CHD: chronic heart disease; A-fib: atrial fibrillation; T2DM: type 2 diabetes mellitus; CKD: chronic kidney disease; GFR: glomerular filtration rate.
Significant findings in **bold** text.
