# Supplementary material for: Influence of drugs on blood potassium levels in older, multi-medicated patients – results of two cohort studies focusing on adverse drug reactions
Source: BMC Geriatr. 2026 Jun 27;26:881. doi: 10.1186/s12877-026-07899-5 (PMC13317038; doi:10.1186/s12877-026-07899-5)
Supplement: Supplementary file 3 — Supplementary Material 3. [file 12877_2026_7899_MOESM3_ESM.docx]

**Supplement 3**. Distribution of drug use in patients with low, normal and high potassium levels (N=1,097) stratified to datasets.

|  | **ADRED-study, n=1011** | | | | **Polypharmacy consultation hours, n=86** | | | |
| --- | --- | --- | --- | --- | --- | --- | --- | --- |
| **Drugs** | **Potassium ≤ 3.49 mmol/l, n=306** | **Potassium 3.50 – 5.00 mmol/l, n=490** | **Potassium ≥5.01 mmol/l, n=215** | **p-value** | **Potassium ≤ 3.49 mmol/l, n=5** | **Potassium 3.50 – 5.00 mmol/l, n=73** | **Potassium ≥5.01 mmol/l, n=8** | **p-value** |
|  | *Expected low potassium levels* | | |  | *Expected low potassium levels* | | |  |
| Low-ceiling diuretics, n (%) | 92 (30.1) | 63 (12.9) | 25 (11.6) | **<0.001** | 2 (40.0) | 14 (19.2) | 1 (12.5) | 0.268 |
| Thiazides, n (%) | 54 (17.6) | 38 (7.8) | 14 (6.5) | **<0.001** | 0 (0.0) | 6 (8.2) | (12.5) | 0.444 |
| Other low-ceiling diuretics, n (%) | 38 (12.4) | 26 (5.3) | 11 (5.1) | **<0.001** | 2 (40.0) | 8 (11.0) | 0 (0.0) | **0.043** |
| Loop diuretics, n (%) | 171 (55.9) | 256 (52.2) | 140 (65.1) | 0.075 | 2 (40.0) | 38 (52.1) | 4 (50.0) | 0.797 |
| Insulins, n (%) | 34 (11.1) | 74 (15.1) | 33 (15.3) | 0.136 | 2 (40.0) | 12 (16.4) | 3 (37.5) | 0.777 |
| Glucocorticoids, n (%) | 37 (12.1) | 44 (9.0) | 38 (17.7) | 0.109 | 1 (20.0) | 9 (12.3) | 1 (12.5) | 0.750 |
| Osmotically acting laxatives, n (%) | 36 (11.8) | 55 (11.2) | 21 (9.8) | 0.489 | 2 (40.0) | 32 (43.8) | 3 (37.5) | 0.871 |
|  | *Expected high potassium levels* | | |  | *Expected high potassium levels* | | |  |
| MRAs, n (%) | 33 (10.8) | 72 (14.7) | 73 (34.0) | **<0.001** | 1 (20.0) | 14 (19.2) | 2 (25.0) | 0.777 |
| ACE inhibitors/ ARBs, n (%) | 168 (54.9) | 334 (68.2) | 146 (67.9) | **<0.001** | 4 (80.0) | 53 (72.6) | 7 (87.5) | 0.626 |
| ACE inhibitors, n (%) | 90 (29.4) | 188 (38.4) | 78 (36.3) | 0.064 | 2 (40.0) | 23 (31.5) | 3 (37.5) | 0.989 |
| (ARBs), n (%) | 79 (25.8) | 148 (30.2) | 70 (32.6) | 0.086 | 2 (40.0) | 30 (41.1) | 4 (50.0) | 0.676 |
| Beta blocking agents, n (%) | 191 (62.4) | 329 (67.1) | 160 (74.4) | **0.004** | 4 (80.0) | 53 (72.6) | 6 (75.0) | 0.902 |
| NSAIDs, n (%) | 20 (6.5) | 44 (9.0) | 11 (5.1) | 0.705 | 0 (0.0) | 2 (2.7) | 2 (25.0) | **0.014** |
| Coxibs, n (%) | 2 (0.7) | 0 (0.0) | 7 (3.3) | **0.006** | 0 (0.0) | 2 (2.7) | 0 (0.0) | 0.898 |
| Potassium, n (%) | 41 (13.4) | 39 (8.0) | 14 (6.5) | **0.005** | 2 (40.0) | 2 (2.7) | 0 (0.0) | **0.005** |

ARBs: Angiotensin II receptor blockers; MRAs: mineralocorticoid receptor antagonists; NSAIDs: Non-steroidal anti-inflammatory drugs.
Significant findings in **bold** text.
