# Supplementary material for: Influence of drugs on blood potassium levels in older, multi-medicated patients – results of two cohort studies focusing on adverse drug reactions
Source: BMC Geriatr. 2026 Jun 27;26:881. doi: 10.1186/s12877-026-07899-5 (PMC13317038; doi:10.1186/s12877-026-07899-5)
Supplement: Supplementary file 4 — Supplementary Material 4. [file 12877_2026_7899_MOESM4_ESM.docx]

**Supplement 4**. Predictions of changes of serum potassium levels (median Q50 (Q25; Q75)) in mmol/L stratified in single stages of chronic kidney disease (CKD) according to estimated glomerular filtration rates (GFR) in multivariate quantile regression models.

|  | **CKD stage 1, GFR≥90 ml/min/1.73m^2^, n=28** | | **CKD stage 2, GFR 60 - 89 ml/min/1.73m^2^, n=253** | | **CKD stage 3, GFR 30 - 59 ml/min/1.73m^2^, n=456** | | **CKD stage 4, GFR 15 - 29 ml/min/1.73m^2^, n=193** | | **CKD stage 5, GFR ≤15 ml/min/1.73m^2^, n=71** | |
| --- | --- | --- | --- | --- | --- | --- | --- | --- | --- | --- |
|  | ∆Q50 (∆Q25; ∆Q75) | p-value per Q50 (Q25; Q75) | ∆Q50 (∆Q25; ∆Q75) | p-value per Q50 (Q25; Q75) | ∆Q50 (∆Q25; ∆Q75) | p-value per Q50 (Q25; Q75) | ∆Q50 (∆Q25; ∆Q75) | p-value per Q50 (Q25; Q75) | ∆Q50 (∆Q25; ∆Q75) | p-value per Q50 (Q25; Q75) |
| Male sex | 0.44 (0.09; 0.03) | 0.215 (0.834; 0.945) | 0.22 (-0.04; 0.21) | **0.037** (0.755; **0.020**) | 0.36 (0.27; 0.17) | **<0.001** (**0.007**; 0.113) | 0.29 (0.21; 0.43) | 0.164 (0.248; **0.023**) | 0.22 (0.33; -0.31) | 0.662 (0.405; 0.388) |
| Low-ceiling diuretics | -0.17 (-0.35; -0.44) | 0.700 (0.502; 0.497) | -0.63 (-0.74; -0.68) | **<0.001 (<0.001; <0.001)** | -0.50 (-0.40; -0.57) | **<0.001 (0.002; <0.001)** | -0.59 (-0.68; -0.31) | **0.023 (0.003;** 0.185) | -0.02 (-0.34; -0.21) | 0.974 (0.485; 0.678) |
| Loop diuretics | -0.09 (0.42; 0.49) | 0.820 (0.341; 0.361) | -0.39 (-0.42; -0.41) | **0.001** (**0.001;** **<0.001**) | -0.26 (-0.10; -0.22) | **0.013** (0.328; 0.055) | -0.13 (-0.42; -0.37) | 0.599 (0.053; 0.097) | -0.03 (0.06; 0.03) | 0.956 (0.877; 0.938) |
| MRAs | 0.05 (0.14; -0.10) | 0.924 (0.832; 0.902) | 0.71 (0.74; 0.66) | **<0.001** (**<0.001; <0.001**) | 0.26 (0.32; 0.44) | 0.056 **(0.020; 0.003)** | 1.02 (0.97; 0.45) | **<0.001 (<0.001; 0.029)** | -0.19 (0.08; 0.20) | 0.786 (0.975; 0.713) |
| ACE inhibitors/ ARBs | -0.46 (0.03; -0.40) | 0.226 (0.936; 0.455) | 0.17 (0.20; 0.00) | 0.112 (0.088; >0.999) | 0.24 (0.33; 0.21) | **0.023** (**0.001**; 0.063) | 0.37 (0.39; 0.19) | 0.095 (**0.044**; 0.346) | 0.40 (1.06; -0.20) | 0.461 (**0.008**; 0.630) |

ARBs: Angiotensin II receptor blockers; MRAs: mineralocorticoid receptor antagonists.
Models adjusted for sex, use of low-ceiling diuretics, loop diuretics, MRAs, ACE inhibitors/ ARBs, and number of other drugs.
Significant findings in **bold** text.
